# A Caenorhabditis elegans Wild Type Defies the Temperature–Size Rule Owing to a Single Nucleotide Polymorphism in *tra-3*

**DOI:** 10.1371/journal.pgen.0030034

**Published:** 2007-03-02

**Authors:** Jan E Kammenga, Agnieszka Doroszuk, Joost A. G Riksen, Esther Hazendonk, Laurentiu Spiridon, Andrei-Jose Petrescu, Marcel Tijsterman, Ronald H. A Plasterk, Jaap Bakker

**Affiliations:** 1 Laboratory of Nematology, Wageningen University, Wageningen, The Netherlands; 2 Hubrecht Laboratory, Netherlands Institute of Developmental Biology, Utrecht, The Netherlands; 3 Institute of Biochemistry of the Romanian Academy, Bucharest, Romania; Huntsman Cancer Institute, United States of America

## Abstract

Ectotherms rely for their body heat on surrounding temperatures. A key question in biology is why most ectotherms mature at a larger size at lower temperatures, a phenomenon known as the temperature–size rule. Since temperature affects virtually all processes in a living organism, current theories to explain this phenomenon are diverse and complex and assert often from opposing assumptions. Although widely studied, the molecular genetic control of the temperature–size rule is unknown. We found that the Caenorhabditis elegans wild-type N2 complied with the temperature–size rule, whereas wild-type CB4856 defied it. Using a candidate gene approach based on an N2 × CB4856 recombinant inbred panel in combination with mutant analysis, complementation, and transgenic studies, we show that a single nucleotide polymorphism in *tra-3* leads to mutation F96L in the encoded calpain-like protease. This mutation attenuates the ability of CB4856 to grow larger at low temperature. Homology modelling predicts that F96L reduces TRA-3 activity by destabilizing the DII-A domain. The data show that size adaptation of ectotherms to temperature changes may be less complex than previously thought because a subtle wild-type polymorphism modulates the temperature responsiveness of body size. These findings provide a novel step toward the molecular understanding of the temperature–size rule, which has puzzled biologists for decades.

## Author Summary

Biologists are fascinated by variation in body size, which is hardly surprising, considering that the range of body sizes spans orders of magnitude from bacteria to blue whales. Even within species, body sizes can vary dramatically. This intraspecies variation is intriguing because it suggests strong associations between body size and environment. Already in 1847, Bergmann noticed that mammals tend to be larger in colder environments. More recently similar relationships were found for ectotherms, which rely for their body heat on the temperature of their surroundings, where more than 85% of the species studied grew larger at lower temperatures. This phenomenon, dubbed the temperature–size rule, has caused a renewed interest to understand how temperature affects body size. Yet the control of the temperature–size rule remains enigmatic, and the hypotheses proposed have been inconclusive. In this paper the authors show that a single nucleic acid change in one gene is required for regulation of the temperature–size rule in the nematode *C. elegans.* Using protein modelling they also show that this subtle change in DNA decreases the function of the encoded protein. The data suggest that temperature adaptation can be simple and far less complex than previously thought.

## Introduction

For many decades biologists have been intrigued by the relation between body size and temperature. It was discovered that ectotherms—animals that maintain their body temperature by absorbing heat from the surrounding environment such as fish and all invertebrates—reproduce later at a larger size when reared at lower temperatures [1–3]. This phenomenon is known as the temperature–size rule, and nearly 90% of ectothermic species studied so far follow this rule [4]. The magnitude of this phenomenon is illustrated by Azevedo et al. [5] who found a 12% increase in wing and thorax size in Drosophila melanogaster when grown at relatively low temperatures. In the case of the nematode C. elegans (strain Bristol N2), an environmental temperature of 10 °C resulted in adults that were ~33% larger than those grown at 25 °C [6].

About 99.9% of all species are ectothermic, and the temperature–size rule is observed in bacteria, protists, plants, and animals, making it one of the most widespread phenomena in ecology. From the perspective of life-history evolution it is not well understood why growing bigger at lower temperatures is beneficial for organisms. Because this thermal plasticity of body size is taxonomically widespread, the reasons are probably diverse and may vary among groups of organisms. It has been suggested that a large body size is advantageous, because it compensates for delayed reproduction by yielding more offspring [7]. Other explanations may be that a larger body size at maturity enables individuals to produce larger offspring or to provide better parental care [2].

Since body size and temperature are the two most important variables affecting fitness [8,9], many experimental and theoretical attempts have been made to explain the mechanism underlying the temperature–size rule. Essentially, an increase in body size can be achieved by increasing cell number, cell size, or by both. Various studies point at the second (cell size) and the third option (cell size and number) as being the most likely explanation for the observed increase in body size at lower temperatures (*Drosophila spp.* [10–12], yellow dung fly [13], and the nematode C. elegans [6]).

Next to these empirical observations, various models have been proposed that are based on a combination of changes in cell size and number. Biophysical models show that the temperature–size rule is the result of unequal effects of temperature on cell growth and cell division [14]. When the effect of temperature on the rate of division is greater than its effect on the rate of cell growth, the model predicts that a low temperature should lead to a larger body size. Recently, a physiological model was proposed by Atkinson et al. [15], which assumes that temperature induced changes in cell size and number depend on the optimisation of oxygen supply at different temperatures. Yet, these empirical and theoretical findings give little insight into the molecular genetic control of body size at lower temperatures.

Unravelling the molecular mechanism underlying the temperature–size rule is hampered by the fact that temperature affects nearly all biochemical processes in a cell, and in theory growing bigger at lower temperatures may have numerous causes. However, low temperatures also have been shown to induce a number of specific physiological and genetic responses in ectotherms [16]. In D. melanogaster gene expression analysis revealed a senescence marker *smp-30* to be induced by low temperature [17]. Van ‘t Land et al. [18] reported the association of the gene *Hsr-omega* with low temperatures in D. melanogaster. Next to these specific gene responses, an early indicator of low temperature is a transient elevation of the cytosolic calcium concentration [Ca^2+^]_i_. Higher cytosolic calcium levels occur not only in response to a rapid cooling but also to more gradual reductions in temperature, and it is a widespread phenomenon observed in plants [19,20] and ectothermic animals [21–24].

Here we aimed to identify and characterize genes underlying the temperature–size rule in a model ectotherm, the nematode *C. elegans*. C. elegans is a suitable model for studying the molecular control of temperature–body size responses because of its completely sequenced genome, isomorphic growth, and cell constancy, and because nematode life-history traits are easy to observe [25]. We found that wild-type Bristol N2 (designated as N2) grew bigger at lower temperatures and thus complied with the temperature–size rule, whereas wild-type CB4856 (designated as CB) defied the rule. The natural variation in body size response to temperature between CB and N2 was caused by a single mutation F96L in a calpain-like protease TRA-3 encoded by *tra-3*. Homology modelling predicts that F96L is likely to reduce the ability of TRA-3 to bind calcium.

## Results/Discussion

### Wild-Type CB Does Not Comply with the Temperature–Size Rule

**Figure 1:**
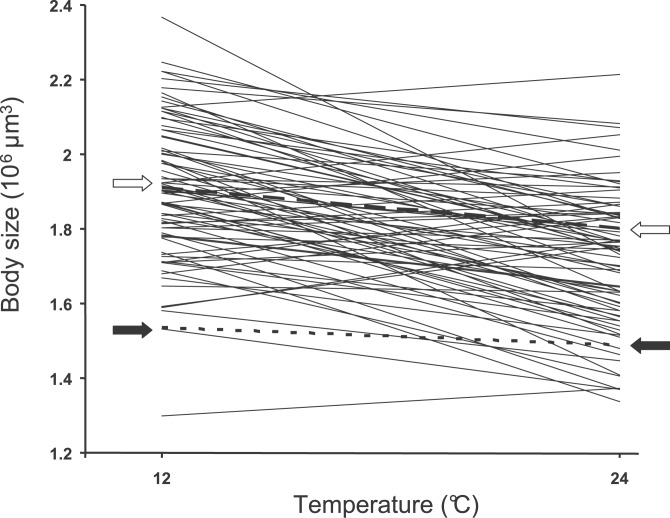
Slope of TRB for 80 RILs and Parental Strains We calculated the slope (10^6^ μm^3^/degree) from a plot of body size versus temperature (12 °C and 24 °C) where: slope = (size at 24 °C − size at 12 °C)/(12 °C − 24 °C). The variance of body size calculated for all the RILs was small and differed only 7% between 12 °C and 24 °C. Most of the lines had a negative slope meaning that at low temperatures individuals grew bigger. Yet 17.5% of the RILs had a positive slope, and a few RILs were nearly nonplastic (i.e., slopes were relatively small). At 12 °C N2 was 35% larger than CB. The TRB for parental strains are designated by arrows: N2, open arrow; CB, solid arrow.

We studied the thermal reaction norm for body size (TRB), which is a plot of body size at maturity versus temperature, and defined compliance with the temperature–size rule if body size is significantly and negatively related to temperature. To assess differences in the TRB between the two wild-type strains we measured body size at 12 °C and 24 °C. Body-size measurements were taken from Gutteling et al. [26]. We found a marked difference in TRB between the two wild types. The body size of wild-type N2 exhibited a significant negative relationship with temperature, i.e., N2 grew larger at low temperature (*F* = 3.49; *p* = 0.02). In contrast, CB defied the temperature–size rule because body size was not significantly affected by temperature (*F* = 0.8; *p* = 0.47) (Figure 1). The results for N2 are in agreement with previous findings where increased body size was found in C. elegans N2 hermaphrodites as well as males at lower temperatures [27,6]. To further study the genetic control of the TRB, we first developed an N2 × CB recombinant inbred panel and performed a quantitative trait locus (QTL) analysis for detecting genomic regions associated with the TRB.

### Large Variation in the TRB among Recombinant Inbred Lines

By selfing the CB × N2 *F*_1_ offspring for 20 generations, we obtained a segregating population of recombinant inbred lines (RILs), which were also exposed to 12 °C and 24 °C. We found large differences in TRB slopes among the RILs (Figure 1). As generally observed in recombinant inbred crosses between divergent strains, the mean trait values for many of the RILs exceeded the mean value for either parental strain. Apparently the differences between the N2 and CB phenotypes (the slope of the TRB) capture a great deal of genetic variation. This was evident in the variation exhibited in the RILs for the TRB slope. Such transgressive segregation has been reported for many organisms and indicates that alleles at different loci act in the same direction, and when combined these alleles will result in phenotypes more extreme than either parent [28].

In general RILs matured at 12 °C at a bigger size than at 24 °C, which is in accordance with the temperature–size rule (see Atkinson [4] for an overview). We found strong genetic variation among RILs for body size across the two temperatures (*F* = 40.1; *p* < 0.001). We then sought to determine which loci were associated with the TRB by genotyping the RILs and performing a QTL mapping study using the recombinant inbred panel.

### Description of the Genetic Architecture of the RILs

For the QTL analysis we used a dense single nucleotide polymorphism (SNP) map. A full description of the genetic architecture of the RILs can be found in [29]. In summary, the overall average distance between two SNP markers was 835 kb or 2.38 cM. The overall average chromosomal coverage was 96% if measured in bp or 95% if measured in cM. Compared to the Wormbase *F*_2_-derived genetic maps (http://www.wormbase.org, release WS106), the genetic maps showed on average an ample 2-fold expansion. This is common for RILs bred by self-fertilization or sib-mating and can be explained by the multiple rounds of meiosis undergone [30].

### Detection of a Specific CB Locus Associated with the TRB on Chromosome *IV*

**Figure 2:**
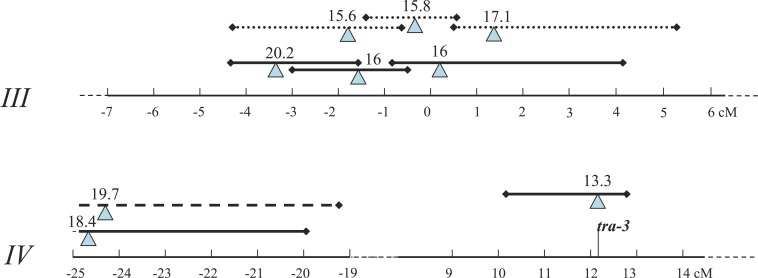
Positions of QTL Associated with Body Size and the Slope of the TRB QTL for body size at different temperatures (12 °C [dotted line] and 24 °C [dashed line]) [26] and the slope of the TRB (solid line) at Chromosomes *III* and *IV.* Triangles are the peak of the QTL and the horizontal bars are CIs. Values near the bars represent the likelihood ratio values. Only those parts of the chromosomes are shown where QTLs were detected.

**Figure 3:**
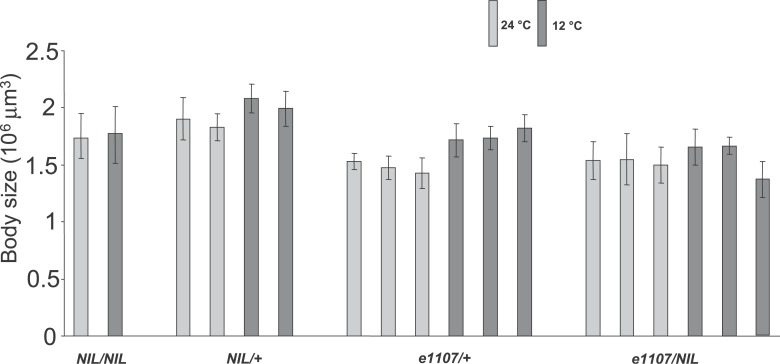
Complementation Analysis: The Effect of Temperature on Body Size in C. elegans Strains and Mutants To test for complementation of F96L we measured body size of the homozygous introgression line NIL WN17–9 (abbreviated as *NIL/NIL*) and heterozygote *F*_1_ obtained from the complementation crosses. Two independent crosses between *NIL* and N2 (*NIL/+*) revealed complementation. Complementation was revealed in three independent crosses of *tra-3(e1107)/+*, whereas three other independent crosses of the heteroallele *tra-3(e1107)/NIL* revealed no complementation. Bars are standard deviation. Significance testing was performed using nested analysis of variance (see Materials and Methods). *NIL/NIL*:*F*_(1,51)_ = 1.54, *NIL/+*:*F*_(1,2)_ = 21.94^*^, *e1107/+*:*F*_(1,4)_ = 59.28^**^, *e1107/NIL*:*F*_(1,4)_ = 4.00, and (* = 0.01 < *p* < 0.05, ** = 0.001 < *p* < 0.01).

Figure 2 shows the detected QTLs associated with the slope of the TRB. Two QTLs on Chromosome *IV* were associated with a negative effect on the slope of the TRB and were linked to CB alleles. The distal QTL at Chromosome *IV* showed pleiotropy or linkage for body size at 24 °C (additive effect of 4%). We aimed to identify the gene(s) controlling the QTL at Chromosome *IV* with a peak at marker pkP4095 at 12 cM, because this QTL was uniquely associated with TRB (hence we named it the TRB-locus) and not with body size itself at 12 °C or 24 °C. This locus had a relatively large additive effect of 34% of the total standard deviation and explained 11% of the among-RIL variance. Introgression of a CB segment spanning the TRB-locus into an N2 background confirmed the QTL analysis. Phenotyping of NIL WN17–9 carrying an ~6-cM region of the TRB locus revealed no significant body-size difference between low and high temperature (Figure 3). Three other QTLs on Chromosome *III* increased the slope and each of these QTLs was linked to N2 alleles and showed a pleiotropic or close linkage effect for body size at 12 °C [26].

### A Promising Candidate Gene within the TRB Locus Is *tra-3*

The 2.5-cM genome segment covered by the confidence interval (CI) of the TRB locus harbours a number of mutationally mapped genes of which only one *(dpy-4)* [31] is known to affect body size. To identify promising candidate genes, we reasoned as follows. Previous studies have shown that body size in C. elegans is controlled by genes that affect cell size and not cell number [32,33]. It is also known that this is one of the main mechanisms, next to cell number, by which ectotherms grow bigger in colder environments [7]. Furthermore, we sought to identify and characterize genes that encode a calcium-activated protein because [Ca^2+^]_i_ is a key signal of low temperature. Lower temperatures lead to an increase of [Ca^2+^]_i_ [21–24].

Given these two facts (increased [Ca^2+^]_i_ and cell size) we searched for genes that are activated by [Ca^2+^]_i_ and that play a role in increased cell size. Among the few genes with known function in the TRB locus, the most likely candidate gene was *tra-3*. TRA-3 has a high homology with mammalian calpains [34], which are known to be activated by [Ca^2+^]_i_ and have been reported to regulate cell size during oncosis (cell swelling) [35]. *dpy-4* is not known to be activated by [Ca^2+^]_i_ [31]. We therefore selected *tra-3* as a candidate gene that might explain the difference in temperature responsiveness between N2 and CB. The gene *tra-3* seems to be important for the TRB slope because a significant difference was found between *tra-3* allelic variants (using the linked marker pkP4095) and the TRB slope (*t*-test, *p* = 0.03). RILs with the N2 allele had a larger slope than RILs with a CB allele.

### Sequencing of *tra-3* in CB and Phenotyping of Artificial *tra-3* Mutants

**Figure 4:**
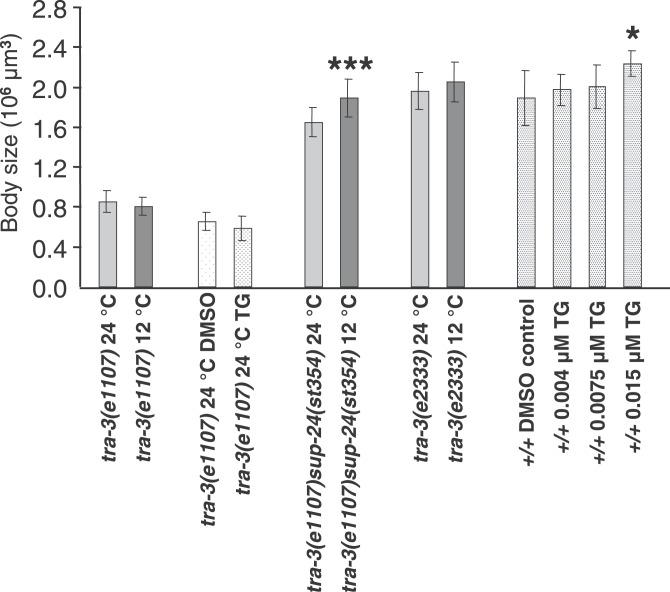
Effect of Temperature and TG on Body Size in C. elegans We measured body size in the mutants *tra-3(e1107)* (size of pseudomales is comparable to normal males but smaller than hermaphrodites) exposed to temperature and TG (including DMSO control). Also shown are the body sizes of the suppressed *tra-3* mutant *tra-3(e1107)sup-24(st354)IV*, and *tra-3(e2333)* exposed to temperature, and wild-type N2 (+/+) exposed to TG and DMSO control. * = significant at 0.01 < p < 0.05 and *** = significant at p < 0.001. Bars are standard deviation. *+/+*:*F*_(1,28)_ = 5.25, *CB*:*F*_(1,47)_ = 0.67, *tra-3(e1107)*:*F*_(1,38)_ = 1.43, *tra-3(e1107)* 24 °C DMSO versus TG:*F*_(1,14)_ = 1.77, *tra-3(e1107)sup-24(st354) F*_(1,22)_ = 14.44, *tra-3(e2333)*:*F*_(1,27)_ = 1.52, and *+/+* 24 °C DMSO versus TG:*F*_(1,8)_ = 6.65.

To investigate the hypothesis that *tra-3* controlled the TRB, we first sequenced this region in CB. One SNP was found within the coding region where phenylanaline-96 in N2 was mutated into leucine-96 in CB. To see whether other *tra-3* mutants displayed the same phenotype as observed in CB, we selected two homozygous artificial allelic mutants in an N2 background, *tra-3(e1107)* carrying a nonsense mutation [34] and *tra-3(e2333)*. We also sequenced *tra-3(e2333)* in the ORF ± 1 kb and found a nonsense mutation at nucleotide position 1,779 (G to A) of the spliced *tra-3* transcript. This resulted in a premature stop (W to stop) at position 593 of the TRA-3 protein. Both mutants were phenotyped for body size at 12 °C and 24 °C and compared to the wild-type N2. Like CB, body size was not affected by temperature in both mutants (Figure 4). The N2 phenotype was rescued by the fully suppressed mutant *tra-3(e1107)sup-24(st354)IV*, which promotes translational readthrough of the *tra-3(e1107)* mutation (Figure 4).

We then tested whether a larger body size could also be obtained by mimicking a low temperature environment through an artificial increase of [Ca^2+^]_i_ at 24 °C. Although TRA-3 does not have a specific EF calcium-binding site in C. elegans, a well-conserved region has been shown to bind calcium [36,37]. We used thapsigargin (TG) to increase [Ca^2+^]_i_ [38,39]. We found a clear dose–response relationship between TG and body size, showing that N2 grew larger at 24 °C at increased levels of TG (Figure 4). A significant increase in size was found at 0.015 μM TG compared to a positive control that included the solvent dimethyl sulfoxide (DMSO). Calpain activity was required for the TG-induced body-size enlargement because treatment with 0.015 μM TG did not result in a larger body size in homozygous *tra-3(1107)* mutants (Figure 4). These results indicate that calcium activation of TRA-3 may be controlling body size at different temperatures.

### No Quantitative Difference in Expression of *tra-3* between N2 and CB

In addition to the F96L mutation, the observed phenotypic differences could be due to differential expression of *tra-3*. Therefore, we performed quantitative RT-PCR experiments on cDNA obtained from N2 and CB at 12 °C and 24 °C. It was found that expression was slightly enhanced at 24 °C in both wild types. There was no significant difference in *tra-3* expression across temperatures between N2 and CB (results not shown). Based on these findings we hypothesised that observed TRB differences between N2 and CB were the result of a polymorphism in *tra-3.*

### Complementation Analysis Confirmed that F96L Resulted in a Flat TRB in C. elegans Wild-Type CB

To further investigate the role of *tra-3* in the wild-type CB, we performed a complementation analysis by crossing the near-isogenic line (NIL WN17–9) with *tra-3(e1107).* Heterozygous *F*_1_ from a cross between NIL WN17–9 and N2 revealed the recessive nature of the CB–TRB allele (Figure 3). The body size for the *e(1107)/+ F*_1_ offspring exhibited increased size at 12 °C indicating that *tra-3(e1107)* was recessive (Figure 3). Complementation analysis in which NIL WN17–9 was crossed with *tra-3(1107)* showed no differences in body size of *F*_1_ between high and low temperature (Figure 3). These results show that *tra-3* is required for regulating body size in response to changing environmental temperatures and that an SNP in *tra-3* is able to reduce this ability. We did not attempt to perform a complementation test between NIL WN17–9 and *tra-3(e2333)* because of the dominant nature of *tra-3(e2333)* over other *tra-3* mutants. Homozygous *tra-3(e1107)* worms show partial masculinisation whereas homozygous *tra-3(e2333)* animals are wild-type hermaphrodites. Heteroalleles of these two mutants are also wild-type hermaphrodites indicating a dominance effect of *e2333* over *e1107* [40].

### Rescuing the Wild-Type N2 *tra-3* Phenotype in CB

**Figure 5:**
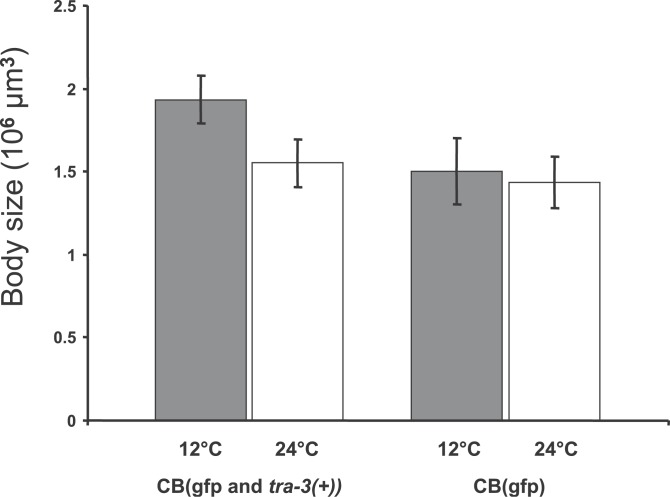
The *tra-3* N2 Allele Makes CB Grow Larger at Low Temperature We measured body size in transgenic CB strains carrying the N2 allele of *tra-3*. Average values are shown for four to five independently derived strains of CB(gfp and *tra-3*(+)) and CB(gfp*)*. The transgenic strains grew significantly larger at low temperature (*t-*test, *n* = 39, *p* < 0.0001). Bars are standard deviation.

We next asked whether the N2 version of the *tra-3* gene could transform CB to have a larger body size at low temperature. Therefore we carried out a transgenic assay in which *tra-3* from N2 was transferred to the CB background. We exposed independently derived strains of CB(gfp) (control strains) and CB(gfp and *tra-3*(+)) to 12 °C and 24 °C. Figure 5 shows that the N2 phenotype was rescued in CB(gfp and *tra-3*(+)) because it grew 24% larger at the low temperature. CB(gfp) retained the CB phenotype because it did not grow larger at low temperature.

### Homology Modelling

**Figure 6:**
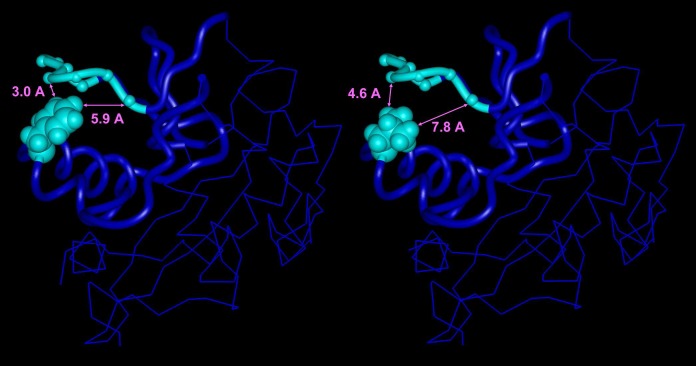
Comparison of the “Open” Configuration of F96 and L96 Variants of the TRA-3 DIIa Domain The region between 61–114 and 149–161, containing the calcium-binding site, and the sequence stretch around position 96 is represented as a solid oval ribbon while the rest is in trace representation. Position 96 and the flexible calcium loop 66–73 with alpha carbons are shown as balls are in light blue. The side chains of N2 F96 (left) and its CB mutant L96 (right) are in space-filling representation. Mutation F96L introduces a void of ~1.5 Å that the loop containing the calcium-binding site can sample during its dynamics. As the number of configurations increases, this might reduce the ability for calcium binding. Distances shown in the figure are between the closest atom of the lateral chain of the F/L96 and the alpha carbons of F66 and G69 respectively.

We next sought to determine whether F96L could lead to a diminished activity of TRA-3 in CB by conducting homology modelling of the 3D structure of TRA-3. The TRA-3 protein consists of four domains (I–III and T), where domain II is the protease catalytic site, and domain T does not have a critical calcium-binding function [34,41] but may be important for protein folding. Although TRA-3 does not have a specific EF calcium-binding site in *C. elegans,* a well-conserved region spanning the boundaries of domain II and III has been shown to bind calcium [36]. In addition, Moldoveanu et al. [37] reported on non-EF calcium-binding sites in domain II between position 62–74 (Ca-1). Homology modelling shows that F96 is located at the beginning of a short helix, H6, contiguous in space to the loop hosting Ca-1. In “open” configuration, corresponding to the absence of calcium, the distance between the α-carbons of F96 and E68, G69, and A70 reduces to 8–10 Å, as compared to ~10–14 Å corresponding to the “closed” configuration. In addition, the side chain of F96 is oriented toward the Ca-1 loop making their atoms to come frequently in van der Waals contact (<3.0 Å) (Figure 6). As the length of a leucine side chain is ~1.5 Å smaller than that of a phenylalanine, F96L will introduce a void in this region. Therefore, F96L can make a small but important difference by increasing the conformational space that the “opened” Ca-1 loop can sample during its dynamics. As the number of configurations increases this might reduce the probability to find the loop in its “closed” configuration and consequently reduce the ability for calcium binding.

### Role of Calcium-Activated TRA-3 in Modulating Body Size

The genetic control of the C. elegans body size has been intensively studied. Mutants such as *sma-2, 3, 4,* and *daf-4* have a small body size and are defective in the TGF-β signalling pathway, which underlies body growth and development [42]. The *lon* mutants have been found to grow longer but not larger in volume [32,43]. It was shown that *egl-4* mutants, defective in a gene encoding a cGMP-dependent protein kinase, have a much larger body size than N2 [32].

Here it is shown that TRA-3 has a prominent role in regulating the thermal plasticity of body size in C. elegans. Homology modelling shows that the F96L mutation in CB4856 attenuates the ability to grow bigger at lower temperatures by destabilizing the calcium-binding site in TRA-3. These data indicate that calcium signalling in response to temperature changes may lead to the activation of TRA-3. This mechanism to control the temperature–size rule is supported by various reports on the elevation of the free cytosolic calcium concentration in response to lower temperatures. Increase of cytosolic calcium levels in response to a gradual reduction of temperature is widely observed in plants [19,20] and ectothermic animals.

Many studies in other organisms have shown the importance of calpains in oncosis showing calpain-mediated cell swelling and disruption of plasma membrane permeability followed by cell death [35]. In C. elegans calcium-activated TRA-3 is known to be involved in the sex determination pathway by activating TRA-2A, a membrane protein that indirectly activates the zinc finger transcriptional regulator TRA-1A by binding and inhibiting a masculinising protein FEM-3 [44]. Current insights are insufficient to link aforementioned findings and to infer a putative pathway by which calcium activation of TRA-3 results in larger cell sizes in C. elegans.

### Do Our Results Corroborate with Existing Theories on the Temperature–Size Rule?

Many different theories have been proposed to unravel the underlying mechanism of the temperature–size rule [2,7,45,46]. Our results partly fit the theory by Van der Have et al. [14] who suggested that the temperature–size rule is regulated by two distinct processes underlying temperature effects on body size: growth rate (which is the biomass increase per time unit) and differentiation rate (which is the reciprocal of development time). Their model presupposes that the temperature–size rule depends on a wide range of alleles differing in sensitivity to temperature. Our results show that a polymorphism in a single gene may attenuate the TRB in C. elegans.

CB was originally isolated in Hawaii while N2 originates from the UK. Whether the F96L mutation in CB reflects adaptive change or a fortuitous event is unknown. Both parental strains have been isolated decades ago and kept in the laboratory ever since, and additional field research is needed to establish whether this polymorphism and/or others in *tra-3* are typical for strains isolated from tropical regions.

Our results do not provide insight into how natural selection modifies the temperature–size rule, yet they provide the basis for a more mechanistic understanding of the evolutionary outcomes. Like C. elegans the increased body size at lower temperatures in flatworms, *Drosophila spp.,* and protists [47–49] is caused primarily by increased cell size. Because *tra-3* shows a high homology with other ectothermic calpains [34,37], our findings may imply a possible role of calpain in the control of the temperature–size rule in other organisms as well.

### Concluding Remarks

We have presented genetic and structural evidence that an SNP in the gene *tra-3* encoding a calpain-like protease is required for the regulation of the temperature–size rule in wild-type *C. elegans.* First, we found that the wild-type N2 complied with the temperature–size rule, whereas wild-type CB4856 defied it, and demonstrated that the genetic variation in the temperature–size response mapped to a single QTL on Chromosome *IV* harbouring *tra-3*. Second, we showed similar expression levels in *tra-3* between the two wild types. Third, transgenic CB carrying an N2 allele of *tra-3* complied with the temperature–size rule. Fourth, we found that F96L in TRA-3 attenuates the ability of wild-type CB4856 to grow larger at low temperatures. Finally, we showed that, based on homology modelling, the CB4856 mutation decreased the calcium-binding activity of TRA-3 rendering it less active. Because TRA-3 shows a high homology with other ectothermic calpains, our findings imply a possible role of *tra-3* in the control of the temperature–size rule in other organisms as well. Together our data show that the response of a quantitative trait to temperature changes can be simple and far less complex than previously thought.

## Materials and Methods

### Strain culturing.

Both N2 and CB parental strains were homozygous. Strains were grown in 9-cm petri dishes at 15 °C or 20 °C on standard nematode growth medium with Escherichia coli strain OP50 as food source [50] and transferred to new dishes by a chunk of agar once a week. RILs were constructed according to [29].

### Development of near-isogenic line by introgression.

NIL WN17–9 was constructed by crossing a single L4 hermaphrodite of RIL WN17 with five males generated from N2 on a 6-cm petri dish. The proportion of males in the offspring was approximately 0.5 indicating a successful cross. Subsequently, 12 crosses were set up, each with the use of single L4 hermaphroditic offspring of the former cross and five males derived from N2. Backcrossing procedure was continued with two L4 hermaphroditic offspring per successful cross. After described three generations backcrossing, six L4 hermaphrodites were picked from each successful cross and placed individually on a 3-cm petri dish to self. Selfing was continued for ten generations for each of the lines. All derived lines were subsequently genotyped at seven marker positions (including marker pkP4095) distributed equally over the fragments that were identified in RIL WN17 to be of CB origin. A total of five lines that appeared to have N2 alleles in all genotyped positions except for the marker pkP4095 were used for detailed genotyping. These lines were genotyped at all remaining marker positions. The results for one of the genotyped lines (NIL WN17–9) showed at all genotyped markers N2 alleles except for CB allele at marker pkP4095, and three neighbouring marker positions at Chromosome *IV* indicating a single ~6-cM DNA fragment of CB origin introgressed into N2 background. Genotyping was according to Li et al. [29].

### Phenotypic experiments of the RILs.

Prior to an experiment, all lines (80 RILs and two parental) were synchronised at room temperature by transferring five adult nematodes to fresh 6-cm petri dishes and allowing them to lay eggs for 3–4 h, after which the nematodes were removed. Eggs were allowed to develop at 20 °C, and three days later synchronisation was repeated to get double-synchronised lines.

### Body-size reaction norms.

Measurements for parental and RIL body size at maturation were taken from Gutteling et al. [26]. Maturation was defined as the moment that the first few eggs are laid and can be easily observed. Because of this, body size at maturity can be precisely measured. For each RIL, three replicate experiments were performed using double-synchronised lines as a start. In each replicate, four adult nematodes per RIL were transferred to a fresh 6-cm dish, allowed to reproduce at room temperature for 2–4 h (average 2.5 h), and removed. Dishes were then stored at 12 °C and 24 °C climate chambers (Elbanton, http://www.elbanton.nl). Temperature was recorded with Tinytag Transit temperature loggers (Gemini Data Loggers, http://www.geminidataloggers.com). After 1 d (24 °C) or 4 d (12 °C), 12 juvenile nematodes were transferred at room temperature to individual dishes (3 cm diameter). Dishes were randomised and put back at the appropriate temperature. After 38 h (24 °C) or 145 h (12 °C) dishes were scanned at room temperature at regular intervals (1.5 h for 24 °C and 4 h for 12 °C) for the presence of eggs. If one or more eggs were observed, time and number of eggs were noted and the dish was put at −20 °C to prevent further development; a pilot study (unpublished data) showed that freezing did not affect body size.

Dishes were defrosted and nematodes were transferred to new dishes with NGM-agar. Digital pictures were taken with a CoolSnap camera (Roper Scientific Photometrics, http://www.photomet.com). Each nematode was measured automatically with Image Pro Express 4.0 (Media Cybernetics, http://www.mediacy.com). Using a measurement ocular we calibrated 10.000 pixels^3^ as 753.516 μm^3^. We assumed a rod-like shape of a worm where volume *V*_szm_ = π·(*D*/2)^2^·*L* = (1/4)·π·*A* ^2^/L where *D* is diameter, *L* is length, and *A* = *L·D*. Because perimeter *P* = 2*L* + 2*D* ~ 2*L* we get:

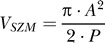



Area (*A*, pixels^2^) and perimeter (*P*, pixels) of each worm were measured digitally. In subsequent analyses *V*_szm_ was used as input value for body size [26]. We studied the TRB, which is a plot of body size versus temperature, and used the slope of the reaction norm as a mapping trait.

For mutant phenotyping the following strains were used for body-size measurements at 24 °C and 12 °C: wild-type Bristol N2 and CB4856 isolate, *tra-3(e2333), tra-3(e1107)/dpy-4(e116)IV,* and *tra-3(e1107)sup-24(st354)IV*. The *tra-3(e1107)/dpy-4(e116)IV* heterozygotes segregate *dpy-4* homozygotes, heterozygotes, and *tra-3(e1107)* homozygote hermaphrodites, which due to maternal effects are phenotypically wild type and segregate pseudomales [51]. We measured body size in these homozygote pseudomales, as well as the homozygote and heterozygote hermaphrodites. Body size was larger only in the hermaphrodites at 12 °C. Body size in the pseudomales was measured after the characteristic male tail [52] was completely formed. Experiments were performed on agar dishes (3 cm diameter) as described above. Samples were not frozen, but body size was measured directly when worms started laying eggs.

Crosses with the mutants and NIL WN17–9 were conducted by transferring J2 stage worms on small agar dishes (3 cm diameter) with three to five males. The worms were allowed to mate at 24 °C after which the females were transferred to new plates thus allowing them to lay eggs for 3–4 h. Mating was considered to be successful if the ratio of males:hermaphrodites was approximately 1:1 in the *F*_1_ hybrids. After this period females were removed and eggs allowed to develop at subsequent high or low temperature. When reproduction started body size was measured as described above.

TG (Sigma, http://www.sigmaaldrich.com) was applied to agar plates dissolved in DMSO. Different concentrations were added in a volume of 200 μl to petri dishes (3 cm diameter) each containing 2 ml of agar (end concentration in the agar: 0.004, 0.0075, and 0.015 μM) and seeded with E. coli. A positive control was included containing 200 μl of DMSO. After 24 h eggs were transferred to each dish and allowed to hatch. The size at maturity was recorded as described above.

The number of replicate worms measured for their body size were at 24 °C (italics) and 12 °C (bold): *tra-3(e1107) 24,* **16**; *tra-3(e1107)* 24 °C DMSO *10; tra-3(e1107*) 24 °C TG, *10; tra-3(e1107)sup-24(st354) 11,* **13**; *tra-3(e2333*) *19,* **10**; +/+ DMSO control *5;* +/+ 0.004 μM TG *6;* +/+ 0.0075 μM TG *6;* +/+ 0.015 μM TG *6;* NIL/+ *11, 15* **16,** **11**; *e1107*/+ *8, 9, 8,* **8, 8, 9**; *e1107*/NIL *10, 16, 10,* **18, 6, 4**; NIL/NIL *22,* **31.**

### Expression study of *tra-3.*

Populations of N2 and CB were bleached (0.5 M NaOH, 1% hypochlorite) to collect synchronized eggs, which were then inoculated into fresh dishes. For each wild-type strain, four replicate dishes of synchronized eggs were kept in each of the two temperatures until maturity was reached. The nematodes were then collected and frozen in liquid nitrogen. Three independent samples were used for each strain and temperature. For each sample, individuals were synchronized and RNA was extracted using the Trizol method. RNA was subsequently purified (with genomic DNA digestion step) with the RNeasy Micro kit from Qiagen (http://www.qiagen.com). RNA concentration and quality were measured with Nano Drop (http://www.nanodrop.com). From each sample 2 μl of RNA were used to obtain cDNA using Superscript II reverse transcriptase from Invitrogen (http://www.invitrogen.com) and oligo d(t) primers from Genisphere (http://www.genisphere.com). cDNA was diluted 20× and used for RT-PCR with iQ Sybr Green Supermix from Bio-Rad in 20 μl reactions (http://www.bio-rad.com). Standard curves for each sample were generated by serial dilutions of the cDNA to select for primer efficiencies of 90%–110% and correlation coefficients greater than 0.99. We selected two reference genes *(rps-20* and *rpl-3)* using geNorm on the basis of Vandesompele et al. [53]. All primers were designed with Beacon Designer avoiding secondary structures and cross homology. RT-PCR runs were done with MyIQ from Bio-Rad, and expression levels were calculated with the Bio-Rad Gene Expression Macro version 1.1 using the selected reference genes for normalization. Expression levels are presented relative to the lowest expression of the gene. At least two independent experiments were carried out for each gene.

### Transgenic strains.

Transgenic worm strains containing *tra-3(+)* from the Bristol N2 wild-type strain in the CB background were obtained from the Umeå Worm/Fly Transgenic Facility (http://www3.umu.se/utcf/index_eng.html). Standard microinjection methods were used [54]. A DNA fragment spanning the entire *tra-3* locus and containing the endogenous *tra-3* promoter was injected at a concentration of 25 μg/ml. The coinjection marker was pCC [55], a plasmid containing gfp under the control of the *unc-122* promoter, which is active in coelomocytes. pCC was injected at a concentration of 50 μg/ml. Body size was measured as described above for five independently derived strains of CB(gfp) (control strains) and CB(gfp and *tra-3*[+]).

### Statistical analysis.

For RIL analysis a randomised block design was used (three blocks per RIL). Statistical analyses were performed in SAS. All data were found to be normally distributed according to the Box-Cox method. Comparison between treatments was tested with one-way ANOVA using PROC MIXED. In case of crossing experiments, replicate crossings were performed, and the data were analysed with a nested design where each cross was nested within temperature (cross[temperature]). In PROC MIXED we defined cross(temperature) as a random factor. The number of replicates was optimal to obtain the mean to be within the 95% CI. ANOVA was performed to study the effect of temperature, RIL, block, and interactions on body size. QTL mapping was used to identify the genomic regions (Wormbase release WS100) controlling various life-history traits. Composite interval mapping was used to identify responsible QTL because it is statistically a well-established and powerful tool; it has a better resolution of QTL peaks compared to interval mapping and is able to control for a number of background markers [56]. QTL analyses were performed with the software package QTL Cartographer version 2.0 [57] using forward regression, a maximum of five background parameters, and the default window size of 10 cM. The experiment-wise likelihood-ratio threshold significance level was determined by computing 1,000 permutations of each trait [58] as implemented by QTL Cartographer. These permutations can account for non-normality in marker distributions and trait values. A peak in the likelihood ratio LR was taken to indicate a significant QTL if LR > 10. Composite interval mapping is sensitive to the number of background markers included in the analysis. The relatively low number of five background markers was used because too many background markers can over-parameterise the model. However, in order to assess whether detected loci that were close to one another also suggested one QTL, we examined the inclusion of ten background markers. The results show the significant QTL based on this ten-marker correction. CIs for QTL were taken based on a 1-LOD support interval corresponding to 95% CI [59].

### Homology modelling.

Template identification was performed with 3D-PSSM [60]. Results show that the first part of TRA-3 sequence corresponding to domains I–III best matches rat calpains M and μ with E-values of 0.0142 and 0.0332 respectively, corresponding to over 95% fold recognition confidence. By contrast, the last part corresponding to the T domain matches the C2 domain fold with a best hit to protein kinase C alpha C2, E-value: 0.583, corresponding to 90% confidence.

Restricting to only the TRA-3 DII domain where the F96L mutation occurs (Figure S1), best templates were searched for the “open” and “closed” configurations—corresponding to calcium free and bound states respectively.

For the “open” configuration the best match is with human M-calpain (pdb code—1kfu) with an E-value of 0.000686, 36.5% identity, and 67.5% similarity. However this calpain was not crystallized in its “closed” configuration as well, and further search for templates was needed to model this state.

Structural analysis of the existing M and μ calpains crystallized in “closed” state (1tl9, 1mdw, 1kxr, 1tlo, 1zcm, 2ary) showed that all of these are practically identical from a structural point of view, with main chain rms deviations of only 0.731–1.124 Å. Consequently all are equally good templates for TRA-3 DII and the closest sequence homologue can be used. This was found to be the rat μ-calpain (pdb code – 1tl9, identity: 38.6%; similarity: 67.5%, but an E-value of only 0.0553).

In building the models, target and template sequences were first aligned using MULTALIN [61]. This alignment was further optimised manually in several steps by incorporating information on secondary structure, accessibility, contacts, and functionality of important residues. Secondary structure profile of the target was raised by a consensus based on the top five prediction methods according to CASP6 (Critical Assessment of Structure Prediction Methods 6): JPRED [62], HNN [63], SSPRO [64], PROF [65], NNPREDICT [66]. The alignment was further refined by moving the gaps to correct for unfavourable exposures in the 3D model.

The 3D models were then built by coordinate transfer in the sequence conserved regions. Loops with insertions or deletions were generated ab initio, then subjected to multiple rounds of conformational search by simulated annealing and local minimization. Packing of long insertions was investigated using Modeller [67] by generating large number of loop conformers and subjecting them to statistical analysis. Simulated annealing was then used to optimise the top contenders followed by extensive rounds of energy minimisation. In the end, the entire model was subjected to repeated rounds of minimization to relieve unfavourable contacts.

Model building, refinement, and analysis were performed using the Accelrys programs: Insight II, Discover, Homology, Modeller, Charmm, Cdiscover, and the free-ware 8v1 version of Modeller on an Silicon Graphics, Octane 2 station.

## Supporting Information

Figure S1 The Refined Sequence Alignment of the TRA-3 DII with the Two Templates Used for Modelling the “Open” and “Closed” States Respectively (41 KB DOC)

## Abbreviations

CB: CB4856
CI: confidence interval
DMSO: dimethyl sulfoxide
N2: Bristol N2
QTL: quantitative trait locus
RIL: recombinant inbred line
SNP: single nucleotide polymorphism
TG: thapsigargin
TRB: thermal reaction norm for body size
