# Supplementary material for: A Caenorhabditis elegans Wild Type Defies the Temperature–Size Rule Owing to a Single Nucleotide Polymorphism in tra-3
Source: PLoS Genet. 2007 Mar 2;3(3):e34. doi: 10.1371/journal.pgen.0030034 (PMC1808073; doi:10.1371/journal.pgen.0030034)
Supplement: Supplementary file 1 [file pgen.0030034.sg001.doc]

```
Tra3DII-calpains sequence alignment. 1kfu: human M-calpain; 1tl9: rat μ-calpain. L – F96L mutation.
      ▲ - residues involved in Ca binding
```

**⊗ - residues of the active site**

```
SS1kfu & SS1TL9 are the secondary structure assignments from the 1kfu & 1TL9 crystal structures; SStra3 is the secondary structure predicted for the tra3 sequence
Highlighted in green are sequence regions with identity matches, highlighted in yellow are similarity matches


        1        10        20        30        40             50        60        70        80        90        100       110
        |--------|---------|---------|---------|--------------|---------|---------|---------|---------|---------|---------|--
SS1kfu: CCCCCCCEEHHHCCHHHHHHHHHHHCCCCCCCCCCCCCCCCCCCCCCCCCCCCCCEEEECHHHHCCCCECCCCCCCCCCEECCCCCCHHHHHHHHHCCCCHHHHCCCCC----CCCC
SS1tl9: -----CCEEHHHCCHHHHHHHHHHCCCCCCCCCCCCCHHHHCCCCCCCCCHHHCCCEEECHHHHCCCCECCCCCCCHHHCECCCCCCHHHHHHHHHHCCCHHHHHHHCC----CCCC
>1KFU   LGSHERAIKYLNQDYEALRNECLEAGTLFQDPSFPAIPSALGFKELGPYSSKTRGMRWKRPTEICADPQFIIGGATRTDICQGALGDCWLLAAIASLTLNEEILARVVP----LNQS
>1TL9   LGRHENAIKYLGQDYENLRARCLQNGVLFQDDAFPPVSHSLGFKELGPNSSKTYGIKWKRPTELLSNPQFIVDGATRTDICQGALGDCWLLAAIASLTLNETILHRVVP----YGQS
>tra3   MTRSEKTRHFGNQNYEKLRKICIKKKQPFVDTLFPPTNQSLFLEQ-----RQSSDIVWKRPGELHPDPHLFVEGASPNDVTQGILGNCWFVSACSALTHNFKLLAQVIPDADDQEWS
SStra3: CCCCCCCCCCCCCCHHHHHHHHHHCCCCCCCCCCCCCCCCCCCCC-----CCCCCEEEECCHHHCCCCCEEECCCCCCCEECCCCCCHHHHHHHHHHHHCHHHHHHCCCCCCCCCCC
```

**▲▲▲ ▲ ▲ ⊗	⊗	L**

```
               120       130       140       150       160       170       180       190       200       210       220       230
        -------|---------|---------|---------|---------|---------|---------|---------|---------|---------|---------|---------|--
SS1kfu: CCCCCCCEEEEEEECCCCEEEEEEECCEEEECCEECCCEECCCCECHHHHHHHHHHHCCCCHHHCCCCCHHHHHHHCCCCEEEEEECCCCCCC--------HHHHHHHHHCCCCEEEEEC
SS1tl9: CCCCCCCEEEEEEEECCEEEEEEEECCEEEECCEECCCECCCCCECHHHHHHHHHHHHHCCCHHHCCECHHHHHHHHHCCCEEEEEHHHCCCC--------HHHHHHHHHHHCCEEEEEC
>1KFU   FQENYAGIFHFQFWQYGEWVEVVVDDRLPTKDGELLFVHSAEGSEFWSALLEKAYAKINGCYEALSGGATTEGFEDFTGGIAEWYELKKPPPN--------LFKIIQKALQKGSLLGCSI
>1TL9   FQEGYAGIFHFQLWQFGEWVDVVVDDLLPTKDGKLVFVHSAQGNEFWSALLEKAYAKVNGSYEALSGGCTSEAFEDFTGGVTEWYDLQKAPSD--------LYQIILKALERGSLLGCSI
>tra3   TKHAYAGIFRFRFWRFGKWVEVVIDDLLPTRDGKLLFARSKTPNEFWSALLEKAFAKLYGCYENLVGGHLSDALQDVSGGVAETLHVRKFLKDDPNDTELKLFNDLKTAFDKGALVVAAI
SStra3: CCCCCCCEEEEECCECCCEEEEEEECCCCCCCCEEEEEEECCCCHHHHHHHHHHHHHHCCCCHHCCCCCHHHHHHHCCCCCEEEEECCCCCCCCCCCCHHHHHHHHHHHHHCCCEEEEEE
                                               ▲    ▲


               240       250       260       270       280       290       300       310       320       330       340       350
        -------|---------|---------|---------|---------|---------|---------|---------|---------|---------|---------|---------|-
SS1kfu: CCCCCCCCCCCCCCCCCCCCCEEEEEEEEECCCC--------------CCEEEEEEECCCCCCCCCCCCCCCCCCCCCCCCHHHHCC-----CCCCCCCCCCCEHHHHHCCCEEEEEEC
SS1tl9: CCCCHHHCCCECCCCCECCCCEEEEEEEEEEECC--------------EEEEEEEEECCCCCCCCCCCCCCCCHHHHHCCHHHHHHH-----CCCCCCCEEEEEHHHHHHHCCEEEEEC
>1KFU   DITSAADSEAITFQKLVKGHAYSVTGAEEVESNG--------------SLQKLIRIRNPWGEVEWTGRWNDNCPSWNTIDPEERERL-----TRRHEDGEFWMSFSDFLRHYSRLEICN
>1TL9   NISDIRDLEAITFKNLVRGHAYSVTDAKQVTYQG--------------QRVNLIRMRNPWGEVEWKGPWSDNSYEWNKVDPYEREQL-----RVKMEDGEFWMSFRDFIREFTKLEICN
>tra3   AARTKEEIEESLDCGLVKGHAYAVSAVCTIDVTNPNERSFTSFIMGSKRKQNLIRLQNPWGEKEWNGAWSDDSPEWQNVSASQLSTMGVQPANSDSDDGDFWMPWESFVHYFTDISLCQ
SStra3: CCCCHHHHHHHHHCCCCCCCCEEEECEEEEEECCCCCCEEEEEEECCCCCEEEEEEECCCCCECCCCCCCCCCCCCCCCCHHHHHHCCCCCCCCCCCCCCEEECHHHHHHHCCCEEEEC
```

**⊗	⊗	▲	▲	▲ ▲ ▲ ▲**
